# *In vivo* optogenetic tracing of functional corticocortical connections between motor forelimb areas

**DOI:** 10.3389/fncir.2013.00055

**Published:** 2013-04-01

**Authors:** Riichiro Hira, Fuki Ohkubo, Yasuhiro R. Tanaka, Yoshito Masamizu, George J. Augustine, Haruo Kasai, Masanori Matsuzaki

**Affiliations:** ^1^Division of Brain Circuits, National Institute for Basic BiologyOkazaki, Japan; ^2^CREST, Japan Science and Technology AgencySaitama, Japan; ^3^Laboratory of Structural Physiology, Center for Disease Biology and Integrative Medicine, Graduate School of Medicine, University of TokyoTokyo, Japan; ^4^Department of Basic Biology, School of Life Science, The Graduate University for Advanced Studies (SOKENDAI)Myodaiji, Okazaki, Japan; ^5^Center for Functional Connectomics, Korea Institute of Science and TechnologySeoul, Republic of Korea; ^6^Program in Neuroscience and Behavioral Disorder, Duke-NUS Graduate Medical SchoolSingapore

**Keywords:** motor cortex, Channelrhodopsin-2, optogenetics, corticocortical connections, photostimulation mapping

## Abstract

Interactions between distinct motor cortical areas are essential for coordinated motor behaviors. In rodents, the motor cortical forelimb areas are divided into at least two distinct areas: the rostral forelimb area (RFA) and the caudal forelimb area (CFA). The RFA is thought to be an equivalent of the premotor cortex (PM) in primates, whereas the CFA is believed to be an equivalent of the primary motor cortex. Although reciprocal connections between the RFA and the CFA have been anatomically identified in rats, it is unknown whether there are functional connections between these areas that can induce postsynaptic spikes. In this study, we used an *in vivo* Channelrhodopsin-2 (ChR2) photostimulation method to trace the functional connections between the mouse RFA and CFA. Simultaneous electrical recordings were utilized to detect spiking activities induced by synaptic inputs originating from photostimulated areas. This method, in combination with anatomical tracing, demonstrated that the RFA receives strong functional projections from layer 2/3 and/or layer 5a, but not from layer 5b (L5b), of the CFA. Further, the CFA receives strong projections from L5b neurons of the RFA. The onset latency of electrical responses evoked in remote areas upon photostimulation of the other areas was approximately 10 ms, which is consistent with the synaptic connectivity between these areas. Our results suggest that neuronal activities in the RFA and the CFA during movements are formed through asymmetric reciprocal connections.

## Introduction

The coordinated activities of motor cortex network are thought to be essential for elaborate movements. There are two spatially segregated motor forelimb areas in rodents: the rostral forelimb area (RFA) and the caudal forelimb area (CFA), which can be identified by intracortical microstimulation (ICMS) (Neafsey et al., 1986; Rouiller et al., 1993; Tennant et al., 2011) or Channelrhodopsin-2 (ChR2) photostimulation mapping (Hira et al., 2013). When rodents participate in reaching, grasping, and lever-pulling tasks, the RFA and the CFA show similar neuronal activities (Hyland, 1998; Hira et al., 2013). Nevertheless, the largest peaks of neuronal activities in the RFA and the CFA occurred before reach onset and around the time of reach end, respectively (Hyland, 1998). To understand how such activities in the RFA and the CFA are coordinated during movement, it needs to clarify synaptic connections between the RFA and the CFA.

By using anterograde and retrograde tracers, Rouiller et al. (1993) found that neurons in layers above the cluster of the corticospinal neurons of the CFA have dense projections to layers 5 (L5) and 6 (L6) of the RFA, while neurons in L5 and L6 of the RFA project to all layers of the CFA. Projections from lower to higher sensory areas originate from neurons predominantly located in layer 4 (L4) and layer 2/3 (L2/3), while projections from higher to lower sensory areas originate from neurons located in L5 (Van Essen and Maunsell, 1983; Coogan and Burkhalter, 1990; Shipp, 2005). Thus, it is thought that the RFA is a higher motor area than the CFA, although both areas lack L4 (Neafsey et al., 1986; Rouiller et al., 1993; Smith et al., 2010; Tennant et al., 2011). However, whether activation of the RFA induces the CFA activity, or vice versa, has not yet been examined. Recently, both we and another group reported that scanning of light beams across the surface of the cortex can induce cortical activity around the photostimulated points in transgenic mice that express ChR2 mainly in layer 5b (L5b) pyramidal neurons (Ayling et al., 2009; Hira et al., 2009). With this optogenetic approach, the motor areas where photostimulation induces limb movements can be rapidly and reproducibly mapped. Furthermore, another study used this ChR2-photostimulation mapping method, combined with whole-cortical voltage-sensitive dye imaging, to reveal the output regions of each stimulated cortical area (Lim et al., 2012). However, the functional connections between the RFA and the CFA have not yet been examined by any method. In addition, in mice that express ChR2 in L5b the projections originating from upper layers [L2/3 and layer 5a (L5a); Hooks et al., 2013] cannot be examined. In this study, we circumvented this issue by combining *in vivo* ChR2-based photostimulation mapping with *in vivo* electrical recording of neurons. This was done in either ChR2 transgenic mice, where the L5b neurons express ChR2 (Wang et al., 2007), or in mice where both the upper layers and L5b were transfected with an adeno-associated virus (AAV) that encoded ChR2-EYFP (Yizhar et al., 2011). We found that L5 neurons in the RFA receive functional projections from L2/3 and/or L5a, but not L5b, of the CFA. Conversely, L5 neurons in the CFA receive functional projections from L5b of the RFA.

## Materials and methods

### Animal preparations

C57BL/6 and C57BL/6 (Thy1-ChR2-EYFP) mice [line 9; (Arenkiel et al., 2007; Wang et al., 2007)] aged 4–6 months were used in this study. For photostimulation, mice were anesthetized by intraperitoneal injection of ketamine (74 mg/kg) and xylazine (10 mg/kg) and then an incision was made in the skin that covered the neocortex. Once the exposed skull was cleaned, a head plate was attached to the skull with dental cement (Fujiryu-to; GC, Tokyo, Japan). Mice were then either subjected to experimentation immediately after the surgery or were allowed to recover for several days. In cases where the animals were given time to recover, the surface of the intact skull was coated with clear acrylic dental resin (Super bond; Sun Medical, Shiga, Japan) to prevent drying (Hira et al., 2009). The animal experimental committee of the School of Medicine, University of Tokyo and the Okazaki animal committee approved all experiments in this study.

### Intracortical microstimulation (ICMS)

ICMS was performed as described (Hira et al., 2009, 2013). Briefly, wild-type mice were anesthetized by intraperitoneal injection of ketamine (74 mg/kg) and xylazine (10 mg/kg). The area of the skulls that covered either the left or the right cortical hemisphere was then removed. Tungsten or elgiloy microelectrodes (WPI, Sarasota, FL) were inserted to a depth of more than 0.6 mm beneath the cortical surface. The cortex was then stimulated with a 45 ms train of 0.4 ms cathodal pulses of 30–200 μA at 333 Hz.

### Virus injections

ChR2 was introduced into neurons in wild-type mouse brains by intracortical injections of rAAV2/9-Syn-hChR2 (H134R)-EYFP (AAV-ChR2-EYFP; 8.58 × 10^12^ vector genomes/ml; provided by the University of Pennsylvania Gene Therapy Program Vector core), as previously described (Masamizu et al., 2011). Mice were anesthetized with either isoflurane (0.8–1.1% for induction and maintenance) or ketamine (74 mg/kg) and xylazine (10 mg/kg), and then 0.5–2.0 μl of virus solution was injected into one, two, or three sites at depths of 300–500 μm below the pia in either the RFA (2.5 mm rostral; 0.8 mm lateral from bregma, left hemisphere) or the CFA (0.2 mm rostral; 1.2 mm lateral from bregma, left hemisphere) through a glass pipette (20–30 μm open tip diameter) at 8–10 psi pressure (T25-15-900; Toohey Spritzer, Fairfield, NJ). The mice were then returned to their homecages and were allowed to recover for more than 2 weeks. This recovery period allowed for sufficient levels of ChR2 to express.

### Optogenetic tracings

ChR2 transgenic and AAV-ChR2-EYFP transfected mice with headplates were anesthetized with isoflurane (0.7–1.1%). The skull that covered either the left or right cortical hemisphere (including both the stimulation and recording sites) was then removed. Photostimulation was performed with a blue laser at 473 nm using an upright microscope (BX61WI; Olympus, Tokyo, Japan) and a FV1000-MPE laser-scanning microscope system (Olympus). Either the entire, or just a portion, of the field of view (6.4 × 6.4 mm when using a 2× objective, numerical aperture [NA] of 0.08, PlanApo, Olympus; or 2.6 × 2.6 mm when using a 5× objective, NA of 0.10, MPLan, Olympus) of the cortical surface was divided into two-dimensional pixel arrays. Each pixel was then individually illuminated. The order of illumination was pseudo-randomly programmed (Hira et al., 2009). Assuming that the refractive index of the brain tissue was 1.38 (Binding et al., 2011), and that the laser beam entered the brain with a half-angle of arcsin[0.10/1.38], then the diameter of the laser spot was 29 μm {= 2 × 200 μm tan[arcsin(0.10/1.38)]} at a depth of 200 μm, and 87 μm {= 2 × 600 μm tan[arcsin(0.10/1.38)]} at a depth of 600 μm, from the vasculature. These are lower estimates that do not account for the effects of light scattering. The distance between the center of the mapping area and the recording site in Figure 4 was 2.46 ± 0.27 mm (*n* = 5 sites from which a photostimulation-induced response was detected), which was not significantly different from the distance between the center of the RFA and the center of the CFA determined by ChR2 photostimulation mapping (Hira et al., 2013; 2.39 ± 0.19 mm, *n* = 4, *P* = 0.67; Student's *t*-test). For electrical recordings, tungsten microelectrodes with impedances of 1.5–2 MΩ (TM33B20KT; WPI) were inserted to the RFA or the CFA. As the electrode was inserted, the rate of spontaneous activity suddenly changed from 3.3 ± 2.4 Hz to 12.0 ± 7.3 Hz (8 penetrations) at depths of ~400–500 μm, which presumably corresponded to the border between L2/3 and L5. When the recording was performed in L5, the recording sites were approximately 50–150 μm deeper than the border (524–662 μm from the cortical surface). When the recording was performed in L2/3, the recording sites were searched at a depth of less than 350 μm from the cortical surface, where the rate of spontaneous activity was low. The signals from the electrodes were amplified (DAM-80 amplifier; WPI), filtered at 300–1000 Hz (SIM900; Stanford Research Systems, Sunnyvale, CA), and sampled at 5 kHz (FV1000 system; Olympus). For pharmacological experiments, 1 mM 6-cyano-7-nitroquinoxaline-2,3-dione (CNQX; a competitive AMPA/kainite receptor antagonist; Tocris, Bristol, UK) or 3 μM tetrodotoxin (TTX; a selective blocker of sodium channels that prevents action potentials; Nacalai Tesque, Kyoto, Japan) was dissolved in a solution that contained 125 mM NaCl, 4 mM KCl, and 5 mM HEPES, and was applied directly to the cortical surface. Recodings were made 30 min after the applications of the CNQX or the TTX.

### Immunohistochemistry of AAV-ChR2-EYFP-positive neurons

More than 2 weeks after injection of 0.5 μl AAV-ChR2-EYFP into one site, the mice were deeply anesthetized with ketamine (74 mg/kg) and xylazine (10 mg/kg) and were then transcardially perfused with 40 ml of phosphate buffered saline (PBS) and 40 ml of 4% paraformaldehyde in PBS (Wako, Osaka, Japan). The brains were removed and then post-fixed with the same fixative for at least 12 h at 4°C. The brains were then embedded in 3% agar in PBS and cut into sagittal sections with a thickness of 50 μm. Sections were incubated with a rabbit anti-GFP antibody (Invitrogen, A6455, 1:500) in PBS containing 0.3% Triton X-100 and 1% normal donkey serum (Millipore, S30-100ML) (PBS-XD) for 9–12 h at room temperature. After washed twice (>10 min each) with PBS containing 0.3% Triton X-100 (PBS-X), sections were incubated with a secondary antibody (Invitrogen, A11034, goat anti-rabbit IgG, Alexa Fluor 488 conjugate, 1:200) in PBS-X for 1 h at room temperature. After washing two times with PBS-X, sections were counterstained with red fluorescent Nissl (propidium iodide 536/617, Invitrogen), mounted on glass slides, and coverslipped. Fluorescence was visualized through a fluorescence microscope (BX53F; Olympus) or by confocal laser scanning microscopy (LSM510; Carl Zeiss, Gottingen, Germany). Layer boundaries were determined as the sites where cell body size or density suddenly changed after performing Nissl staining of the same slices. GFP-positive and GFP-negative neurons were determined according to the averaged fluorescent intensity within the Nissl-stained neuronal somata with clearly visible nucleoli.

### Fluorescent tracing using cholera toxin B subunit

Alexa Fluor 594-conjugated cholera toxin B subunit (CTB; CTB-Alexa 594; Invitrogen) was used as a retrograde tracer. The injection of CTB-Alexa 594 was performed, as described before with slight modifications (Tanaka et al., 2011). Briefly, a glass pipette with CTB-Alexa 594 in PBS was inserted into either the RFA or the CFA. Approximately 0.5 μl of the solution was injected through a pipette tip with a diameter at approximately 40 μm and at a pressure of 5–10 psi. One to seven days after injection of the CTB conjugate, mice were deeply anesthetized and transcardially perfused, and the brains were cut into sections, as described above. Sections were incubated with a goat anti-CTB antiserum (List Biological Laboratories, #703, 1:60,000) in PBS-XD overnight at room temperature. After washing twice (>10 min each) with PBS-X, sections were incubated with a secondary antibody (Invitrogen, A11058, donkey anti-goat IgG, Alexa Fluor 594 conjugate, 1:1000) in PBS-XD for 1 h at room temperature. Sections were counterstained with green fluorescent Nissl (NeuroTrace 500/525, Invitrogen), mounted on glass slides, and coverslipped. For ChR2 transgenic mice, the sections were incubated overnight with a mixture of 1/500-diluted rabbit anti-GFP antibody and 1/60,000-diluted goat anti-CTB antibody in PBS-XD. After washing twice (>10 min each) with PBS-X, sections were incubated for 1 h with 1/200-diluted Alexa Fluor 488 conjugated donkey anti-rabbit IgG (Invitrogen, A21206) and 1/1000-diluted Alexa Fluor 594 conjugated donkey anti-goat IgG (Invitrogen, A11058) in PBS-XD at room temperature. Sections were counterstained with deep red fluorescent Nissl (TO-PRO-3 iodide 642/661, Invitrogen), mounted on glass slides, and coverslipped. Fluorescence was visualized through a fluorescence microscope or confocal laser scanning microscopy. The labeled neurons were counted stereologically in confocal stacks (Howard and Reed, 1998) from the most densely labeled part of each of L2/3, L5a, and L5b of the RFA or the CFA.

### Data analyses

All analyses were conducted using Matlab (version 7; MathWorks, MA). Spiking activity was determined to have occurred when at least two successive signals from 0 to 20 ms after the stimulation were less than −4 SD plus the baseline value. Latency was defined as the time from the start of the photostimulation until the start of the spiking activity. In cases where a series of spontaneous activities occurred prior to the photostimulation, these data were removed from the latency analyses.

### Statistics

Data are presented as mean ± SD. Student's *t*-tests were used for statistical comparisons.

## Results

### Determination of RFA and CFA locations by ICMS

**Figure 1:**
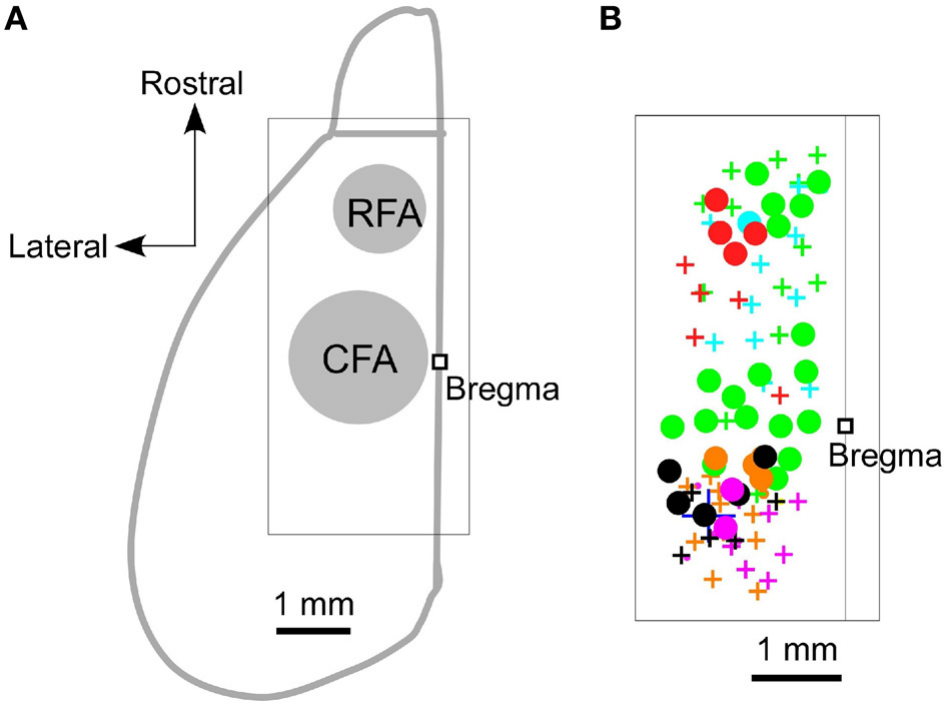
**Locations of the RFA and the CFA. (A)** A schematic dorsal view of the left hemisphere of the mouse neocortex and olfactory bulb. A square was used to denote the location of the bregma. The approximate locations of the RFA and the CFA are labeled and indicated in gray. The boxed region indicates the expanded area shown in **(B)**. **(B)** Circles denote the sites where ICMS induced contralateral forelimb movements, and the crosses denote the sites where the maximum ICMS current injections (200 μA) failed to induce contralateral forelimb movements. Different colors indicate the different animals that were used in these experiments (*n* = 6 mice).

**Figure 2:**
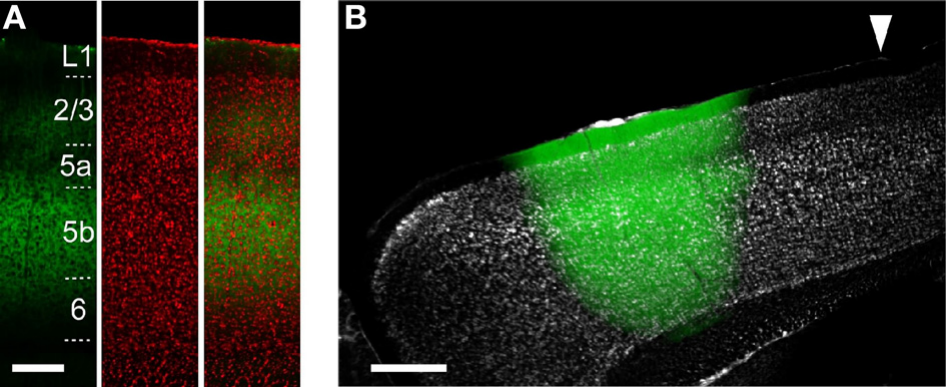
**ChR2 expression in ChR2 Tg and AAV-ChR2 mice. (A)** Anti-GFP immunofluorescence (left), Nissl staining (middle), and the overlay (right) in the CFA in a sagittal section from a ChR2 Tg mouse. Scale bar, 200 μm. **(B)** Overlay of EYFP fluorescence (green) and Nissl staining (gray) in a sagittal section of a mouse in which AAV-ChR2-EYFP was injected into the RFA. Arrowhead indicates 0 mm anterior and 0.8 mm lateral from the bregma. Scale bar, 500 μm.

We began by using ICMS to locate the RFA and the CFA. They were determined to be the areas where ICMS induced contralateral forelimb movements. The rostral area was approximately 2.5 mm rostral and 0.8 mm lateral from the bregma (RFA), and the caudal area was approximately 0.2 mm rostral and 1.2 mm lateral from the bregma (CFA) (Figures 1A,B). This is consistent with our previous study, which used photostimulation mapping in ChR2 transgenic mice expressing a high level of ChR2 in pyramidal neurons in L5b (ChR2 Tg mice; Figure 2A; Wang et al., 2007; Hira et al., 2013). In that study, the center of the RFA and the center of the CFA were approximately 2.5 mm rostral and 1 mm lateral, and 0.5 mm rostral and 1.2 mm lateral, respectively. Throughout the study, we performed electrical recording and injected AAV-ChR2-EYFP based on these coordinate.

### Optogenetic stimulation of cortical neurons

To reveal the functional projections originating from the upper layers (L2/3 and L5a) and L5b, we used two distinct approaches to express ChR2. The first approach was to use ChR2 Tg mice (Figure 2A). The second approach was to use AAV-ChR2-EYFP to express ChR2 in both the upper layers and L5b in the injected region (AAV-ChR2 mice; Figure 2B).

**Figure 3:**
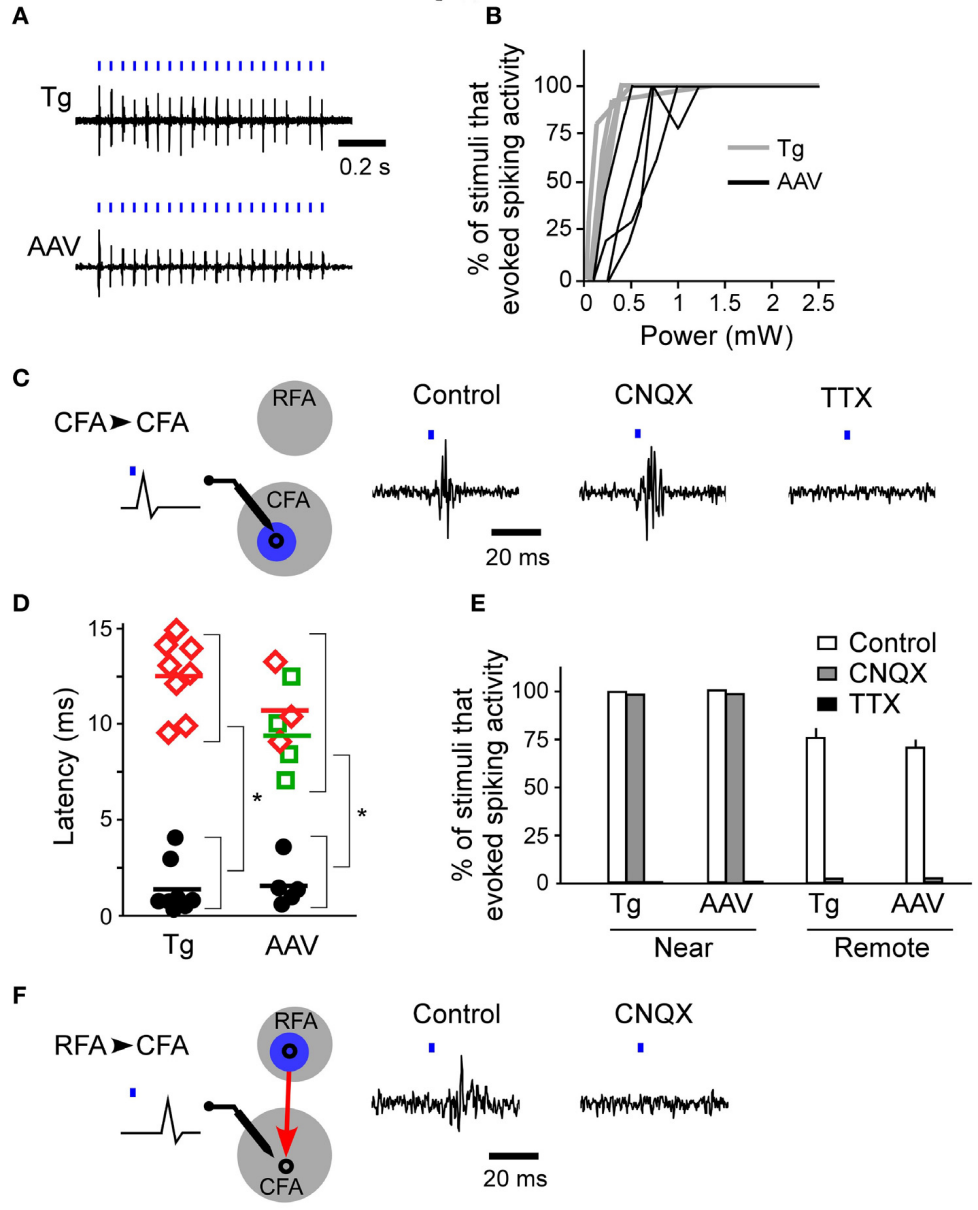
**Firing activities evoked by ChR2 photostimulation of the RFA and CFA. (A)** Representative spiking activities induced by photostimulation of the CFA in the ChR2 Tg and AAV-ChR2 mice. Both traces were recorded in L5 of the CFA. The horizontal distances between the photostimulation points and the recording sites were approximately 200 μm. The blue bars represent the photostimuli (10 ms durations at a frequency of 20 Hz). The laser intensity was 2.5 mW. **(B)** Relationship between the laser power and the percentage of trials in which firing activities were evoked near the photostimulation points (gray, six sites in four ChR2 Tg mice; black, four sites in three AAV-ChR2 mice). The duration of photostimulation was 10 ms. **(C)** Left, A schematic of the experimental arrangement for photostimulation (blue) of the CFA (gray) with simultaneous electrical recording in the CFA. CFA photostimulation directly evoked firing of neurons in the CFA. Right, Representative data showing spike activities recorded in the CFA when the CFA was photostimulated in control conditions, upon CNQX application, and upon TTX application. The data were obtained from the same recording site and the same photostimulation site in a ChR2 Tg mouse. The duration of photostimulation was 2 ms. **(D)** Latencies of evoked spiking activities. Black circles indicate the electrical recordings that were made near the photostimulation points (horizontal distance of approximately 200 μm) in the RFA and the CFA. Red diamonds indicate the electrical recordings in the CFA that were responses to RFA photostimulation. Green squares indicate the electrical recordings in the RFA that were responses to CFA photostimulation. Each colored bar indicates the mean latency for the condition presented in the corresponding color. ^*^P < 10^−4^ by Student's *t*-tests. **(E)** The percentage of trials that evoked spiking activities in control, CNQX application, and TTX application conditions in ChR2 Tg mice (near: control, *n* = 6; CNQX, *n* = 2; and TTX, *n* = 2; remote: control, *n* = 4; and CNQX, *n* = 2) and AAV-ChR2 mice (near: control, *n* = 4; and CNQX, *n* = 2; and TTX, *n* = 2; remote: control, *n* = 4; and CNQX, *n* = 2). Error bars indicate SEM. **(F)** Left, A schematic of the experimental arrangement for photostimulation (blue) of the RFA (gray) with simultaneous electrical recording in the CFA. RFA photostimulation evoked postsynaptic firing activities in the CFA via the synaptic projection (red). Right, Representative data showing evoked activities in the CFA when the RFA was photostimulated. Control conditions and application of CNQX to the CFA are shown. The data were obtained from the same recording site and the same photostimulation site in a ChR2 Tg mouse. The duration of photostimulation was 2 ms.

First, we determined whether blue laser illumination of the cortical surfaces evoked spiking activity in L5 nearest to the illumination points (horizontal distance of less than 300 μm) in both ChR2 Tg mice and AAV-ChR2 mice (Figure 3A). Photostimuli that lasted 2–10 ms induced spiking activities near the photostimulation points in both the ChR2 Tg mice and the AAV-ChR2 mice. Light intensities of ≥1.5 mW induced spiking activities in every trial in both the ChR2 Tg mice and the AAV-ChR2 mice (Figure 3B). The mean onset latencies of spiking activities evoked near the photostimulation points were 1.4 ± 1.6 ms (*n* = 9 sites in 4 mice) for the ChR2 Tg mice and 2.2 ± 0.8 ms (*n* = 5 sites in 3 mice) for the AAV-ChR2 mice (Figures 3C,D). This activity reflected action potentials, rather than some sort of photoelectric artifact, because it was completely inhibited by application of TTX in both lines of mice (Figures 3C,E). Furthermore, spiking activity with short latency was still apparent upon application of CNQX to both the ChR2 Tg and AAV-ChR2 mice (Figures 3C,E). Thus, spiking activity was rapidly induced near the photostimulation points due to direct photostimulation of nearby neurons.

### Optogenetic tracing of functional connections between the RFA and the CFA

**Figure 4:**
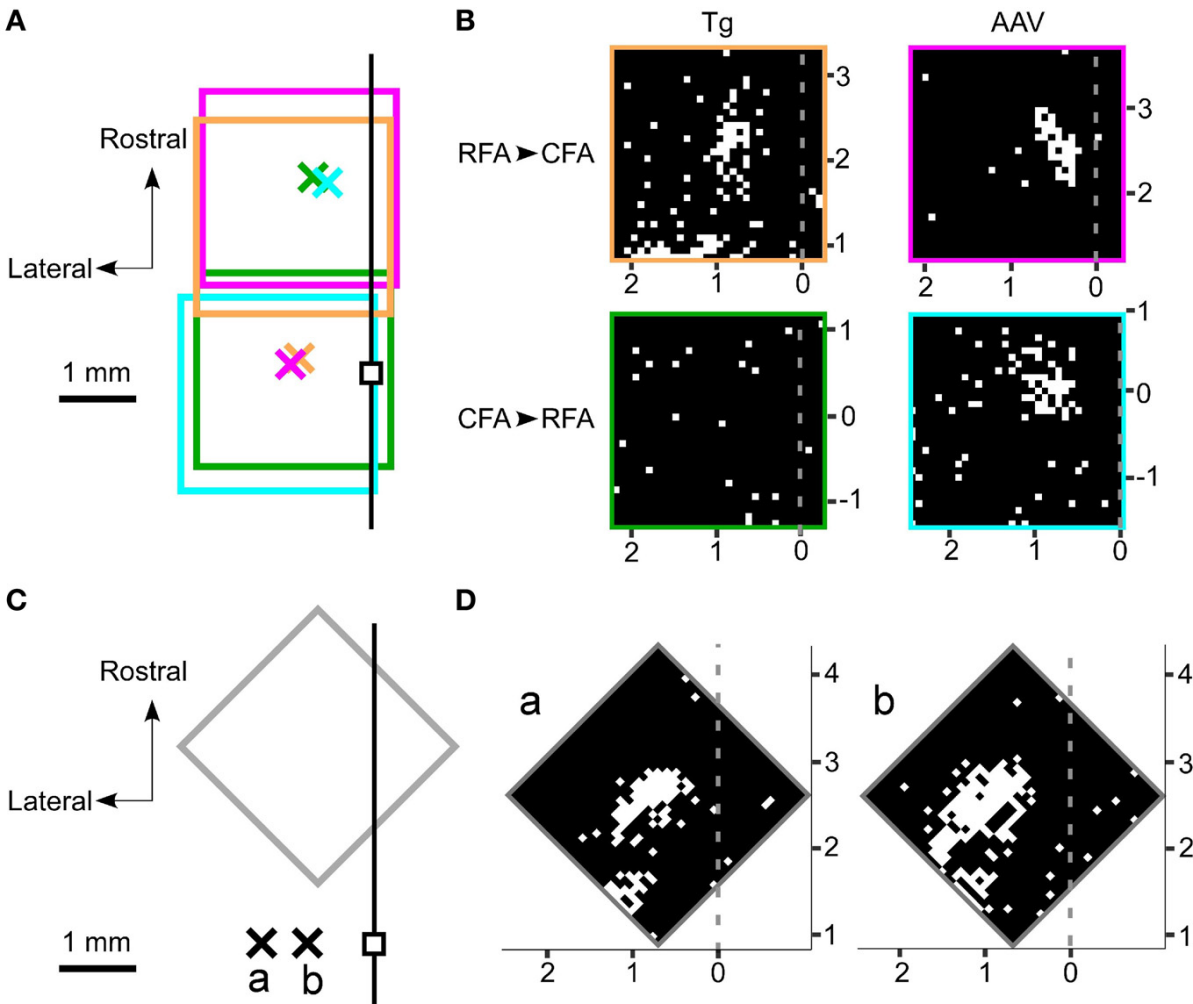
**Photostimulation mapping of functional connections between the RFA and the CFA. (A)** The four rectangles represent the regions mapped by photostimulation. In each region, 32 × 32 points were pseudo-randomly stimulated with a blue laser. The laser intensity was 2.5 mW and the illumination duration was 10 ms. The crosses represent the recording sites. Each color represents one set of recording and photostimulation. The small black square denotes the bregma. The black line represents the midline. **(B)** The laser was used to stimulate 32 × 32 points that are indicated as pixels throughout the RFA and the CFA. White pixels indicate points where photostimulation induced spiking activities and black pixels reflect areas that did not induce spiking activities. The gray dotted lines represent the midline. The mapping was performed in four different mice. The top panels indicate photostimuli that were presented to the RFA while simultaneous recordings were made in the CFA. The bottom panels indicate photostimuli that were presented to the CFA while simultaneous recordings were made in the RFA. The experiments in the left panels were conducted in ChR2 Tg mice, while the experiments in the right panels were conducted in AAV-ChR2 mice. The four areas mapped are indicated by the colored rectangles in **(A)**. **(C)** A gray rectangle indicates the region mapped in **(D)**. The crosses labeled as a and b represent the corresponding recording sites in **(D)**. The recordings were made from the same ChR2 Tg mouse. The black line represents the midline. **(D)** Photostimulation maps of the RFA showed similar patterns of evoked activities even if the recording sites were changed within the CFA. The horizontal distance between the two recording sites shown here was 600 μm.

If the activated neurons near the photostimulation points sent sufficient synaptic projections to a remote area, then the postsynaptic neurons in that remote area should also fire (Figure 3F). Thus, we next assessed whether photostimulation of either the RFA or the CFA induced spiking activity in the other area. We assessed multiple illumination points within and around the RFA and the CFA by simultaneously measuring spiking activity in the other area (Figures 4A,B). Interestingly, photostimulation of the RFA in ChR2 Tg mice reproducibly induced spiking activity in L5 in the CFA (Figures 4A–D). The mean onset latency of responses in the CFA following photostimulation of the RFA was 12.6 ± 1.9 ms (*n* = 8 sites in 4 mice; Figures 3D,F). In contrast, stimulation of the CFA did not induce any detectable spiking in L5 in the RFA of four ChR2 Tg mice (Figure 4B). Thus, there seems to be asymmetry in the functional connections between neurons in the RFA and the CFA. Spiking activity was not detected in L2/3 in either the RFA (two mice) or the CFA (three mice) after photostimulation of the other area.

However, photostimulation of either the RFA or the CFA in the AAV-ChR2 mice induced spiking in L5 in the other area (Figure 4B). The mean onset latencies of spiking activities were 11.0 ± 2.4 ms (*n* = 3 sites in 2 mice) in the CFA in response to photostimulation of the RFA and 9.6 ± 3.1 ms (*n* = 4 sites in 2 mice) in the RFA in response to photostimulation of the CFA (Figure 3D). These latencies of spiking activities (in response to photostimulation of the other area) were significantly greater than the latencies of spiking activities evoked near the photostimulation points (ChR2 Tg mice: *P* = 1.1 × 10^−9^; Student's *t*-test; *n* = 9 sites from four mice for stimulation and recording of the same areas; *n* = 8 sites from four mice for stimulation and recording from different areas; AAV-ChR2 mice: *P* = 8.6 × 10^−5^; *n* = 5 sites from three mice for stimulation and recording from the same areas; and *n* = 7 sites from four mice for stimulation and recording from different areas). By contrast, spiking activity was not detected in L2/3 in either the RFA or the CFA when the other area was photostimulated in AAV-ChR2 mice (two mice for the RFA and two mice for the CFA), although activity was detected in L2/3 near the photosimulation sites (four sites in two mice).

In contrast to the activity evoked near the photostimulation sites, spiking activity in the remote areas did not originate from the neurons that had been directly photostimulated (the antidromically stimulated neurons) because the activities were completely abolished upon application of CNQX (Figures 3E,F). Substantial delays in firing activities (approximately 10 ms) due to synaptically transmitted signals were observed when the CFA was photostimulated and responses were compared in the RFA and the CFA. In addition, similar substantial delays were also apparent when comparing responses in the CFA and the RFA following photostimulation of the RFA. Taken together, these results indicate that neurons in the upper layers, but not L5b, of the CFA induce strong postsynaptic responses in L5 of the RFA, and that RFA neurons in L5b induce strong postsynaptic responses in L5 of the CFA.

### Anatomical tracing of connections between the RFA and the CFA

**Figure 5:**
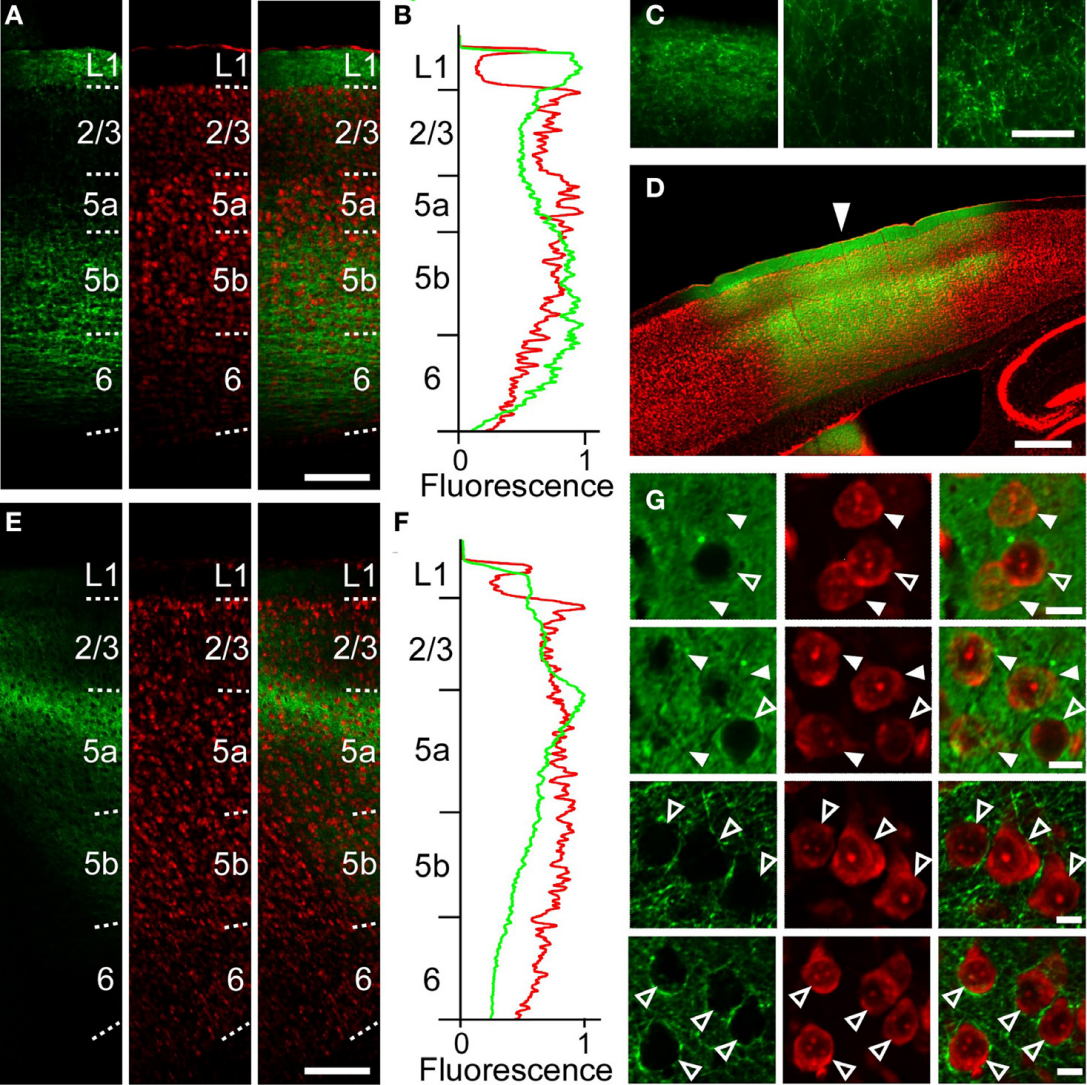
**Anatomical tracings of axonal fibers between the RFA and the CFA. (A)** Anti-GFP immunofluorescence (left), Nissl staining (middle), and the overlay (right) in the CFA in a sagittal section after AAV-ChR2-EYFP injection into the RFA in Figure 2B. Scale bar, 200 μm. **(B)** Profiles of anti-GFP immunofluorescence (green) and Nissl staining (red) in **(A)** as a function of laminar depth. Fluorescence was background subtracted and normalized. **(C)** Higher magnification of GFP-immunofluorescence in **(A)**. Left, L1. Middle, L2/3. Right, L5b. Scale bar, 50 μm. **(D)** Overlay of EYFP fluorescence (green), Nissl staining (red) in a sagittal section of a mouse in which AAV-ChR2-EYFP was injected into the CFA. Arrowhead indicates 0 mm anterior and 1.2 mm lateral from the bregma. Scale bar, 500 μm. **(E)** Anti-GFP immunofluorescence (left), Nissl staining (middle), and the overlay (right) in the RFA in a sagittal section after AAV-ChR2-EYFP injection into the CFA in **(D)**. **(F)** Normalized profiles of anti-GFP immunofluorescence (green) and Nissl staining (red) in **(E)** as a function of laminar depth. **(G)** Higher magnification of the images in **(D)**. Top row, L2/3 at the injection site. Second row, L5a at the injection site. Third row, L2/3 1.2 mm anterior from the injection site. Bottom row, L5a 1.2 mm anterior from the injection site. Closed arrowheads indicate ChR2-EYFP-positive neurons. Open arrowheads indicate ChR2-EYFP-negative neurons. Scale bars, 10 μm.

We next examined whether this asymmetric functional connectivity between the RFA and the CFA was consistent with the anatomical projections between these two areas. For this purpose, we performed anterograde tracing of the AAV-ChR2-EYFP transfected neurons, using an anti-GFP antibody to detect the EYFP-tagged ChR2. After AAV-ChR2-EYFP injection into the RFA (Figure 2B), axonal fibers were densely labeled in layer 1, L5b and upper L6 of the CFA (Figures 5A–C), consistent with a previous report (Smith et al., 2010). AAV-ChR2-EYFP injection into the CFA yielded labeling of axonal fibers densely in L5a of the RFA (Figures 5D–G). The results obtained with the anterograde tracer were consistent with spiking activity occurring in L5, but not in L2/3, when the other area was photostimulated.

**Figure 6:**
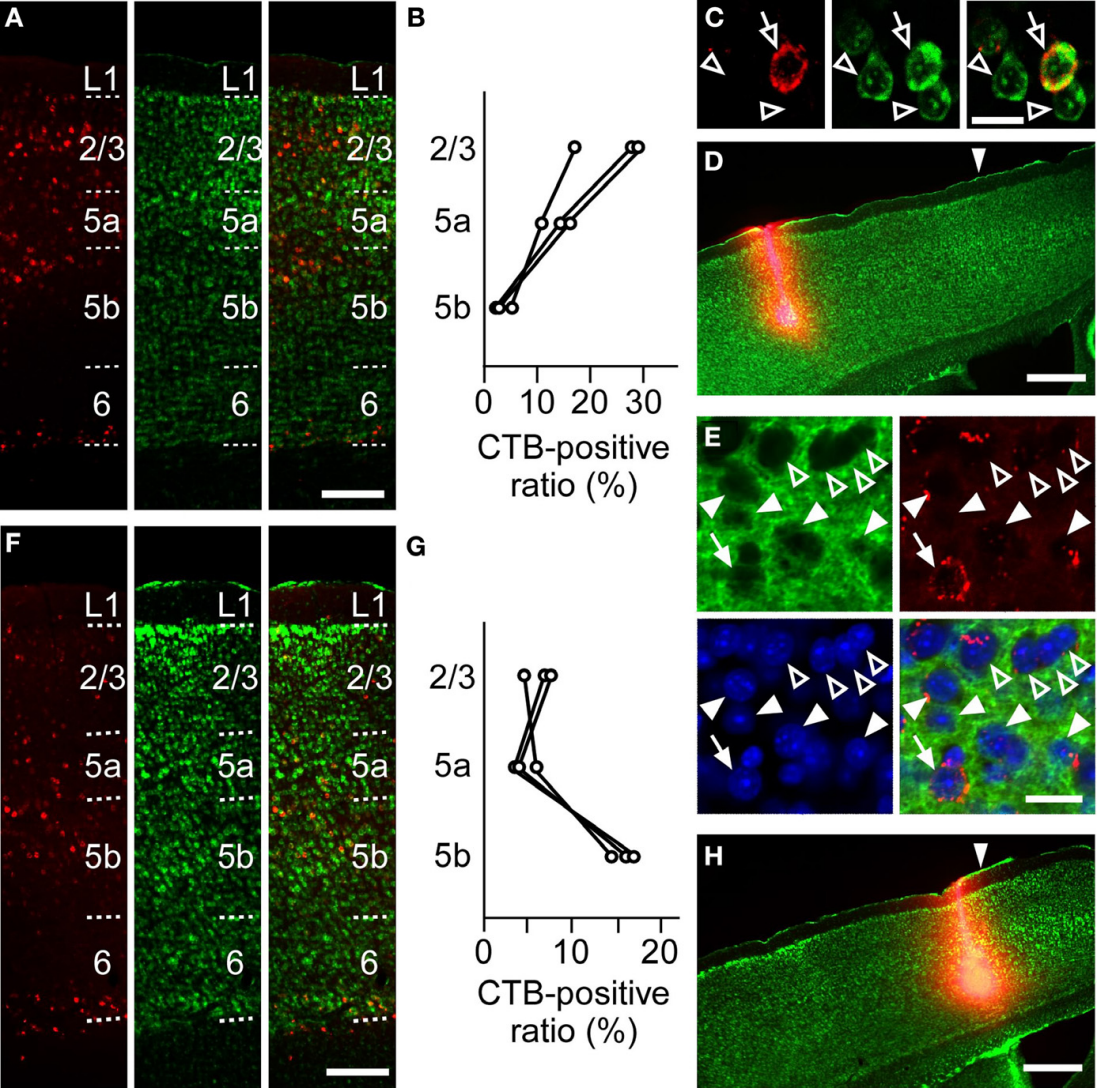
**Anatomical tracings of projection neurons between the RFA and the CFA. (A)** Anti-CTB immunofluorescence (left), Nissl staining (middle), and the overlay (right) in the CFA after CTB-Alexa 594 injection into the RFA in **(D)**. Scale bar, 200 μm. **(B)** Ratios of CTB-positive neurons to Nissl-stained neurons in L2/3, L5a, and L5b of the CFA after CTB-Alexa 594 injection into the RFA. Each line indicates one mouse. **(C)** Higher magnification of the images in L5a in **(A)**. Open arrows indicate a CTB-positive neuron. Open arrowheads indicate CTB-negative neurons. Scale bar, 20 μm. **(D)** Anti-CTB immunofluorescence and Nissl staining of the RFA injected with CTB-Alexa 594 are overlaid. Arrowhead indicates 0 mm anterior and 0.9 mm lateral from the bregma. Scale bar, 500 μm. **(E)** High magnification of the images of anti-GFP immunofluorescence (green), Anti-CTB immunofluorescence (red), Nissl staining (blue), and the overlay in the L5b CFA after CTB-Alexa 594 injection into the RFA of a ChR2 Tg mouse. Closed arrows indicate a ChR2-EYFP-positive and CTB-positive neurons. Closed arrowheads indicate ChR2-EYFP-positive and CTB-negative neurons. Open arrowheads indicate ChR2-EYFP-negative and CTB-negative neurons. Scale bar, 20 μm. **(F)** Anti-CTB immunofluorescence (left), Nissl staining (middle), and the overlay (right) in the RFA after CTB-Alexa 594 injection into the CFA in **(H)**. Scale bar, 200 μm. **(G)** Ratios of CTB-positive neurons to Nissl-stained neurons in L2/3, L5a, and L5b of the RFA after CTB-Alexa 594 injection into the CFA. Each line indicates one mouse. **(H)** Anti-CTB immunofluorescence and Nissl staining of the CFA are overlaid. Arrowhead indicates 0 mm anterior and 1.0 mm lateral from the bregma. Scale bar, 500 μm.

**Figure 7:**
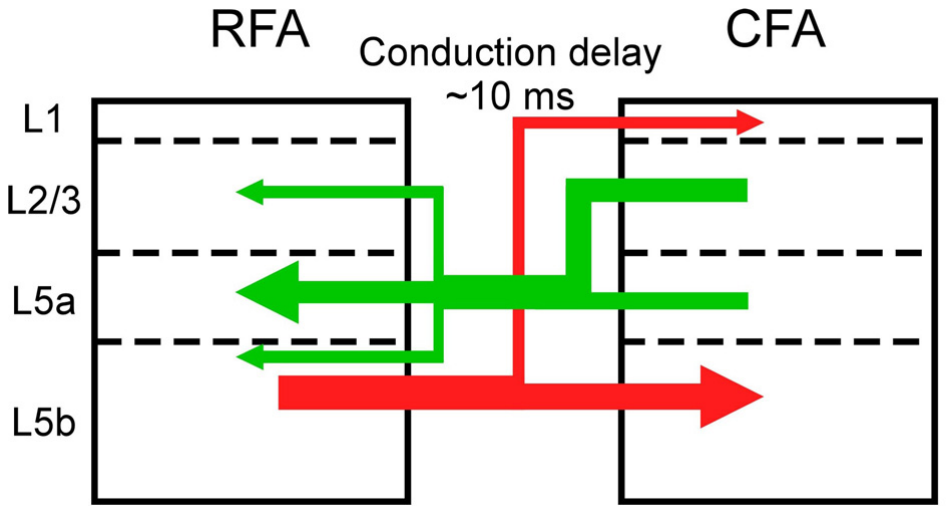
**Asymmetric synaptic connectivity between the RFA and the CFA.** The RFA receives stronger functional projections from L2/3 and/or L5a of the CFA than from L5b of the CFA (green). The CFA receives stronger functional projections from L5b of the RFA than from L2/3 and L5a of the RFA.

To anatomically determine the layer of the projection neurons in the RFA and the CFA, we used CTB subunit as a retrograde tracer. CTB injection into the RFA yielded denser CTB-positive neurons in the upper layers of the CFA than in L5b (the ratio of CTB-positive neurons to Nissl-stained neurons: 24.1 ± 6.2% in L2/3, 13.5 ± 2.9% in L5a, 3.1 ± 1.1% in L5b, *n* = 3 mice; Figures 6A–D). In addition, when CTB was injected into the RFA in ChR2 Tg mice, CTB labeling was detected in 4.3% (3/70) of ChR2-EYFP-positive neurons and 5.1% (3/59) of ChR2-EYFP-negative neurons in L5b of the CFA (Figure 6E). Thus, it was unlikely that a subset of ChR2-EYFP-negative neurons, instead of ChR2-EYFP-positive neurons, in L5b of the CFA strongly projected to the RFA. Thus, the major origin of the projection from the CFA to the RFA was the upper layers. By contrast, after CTB injection into the CFA, the ratio of CTB-positive neurons to Nissl-stained neurons was higher in L5b of the RFA than in the upper layers (6.5 ± 1.0% in L2/3, 4.4 ± 1.4% in L5a, 15.8 ± 1.4% in L5b, *n* = 3 mice; Figures 6F–H). Thus, the dominant direct projection to the CFA originates from L5b of the RFA. All anatomical tracings indicate that the RFA and the CFA communicate with each other through distinct, asymmetric connections (Figure 7).

## Discussion

### Optogenetic tracings of functional connections

In this study, we used an optogenetic mapping method to investigate the connectivity between the RFA and the CFA in mice. Pharmacological experiments and analyses of response latencies demonstrated that spikes evoked in these cortical areas by photostimulation of the other cortical area were postsynaptic responses, rather than directly activated or antidromic action potentials. The transgenic mice that were used in the present study expressed ChR2 in the L5b pyramidal neurons, while AAV transfection introduced ChR2 into neurons in both the upper layers (L2/3 and L5a) and L5b throughout the injected regions. This combination of experiments allowed us to compare projections that originate from L5b to projections that originate from the upper layers and L5b between these cortical areas. In future studies, it will be necessary to limit ChR2 expression to only L2/3 or L5a by using other promoters, such as Wolfram syndrome 1 (Wfs1) or ETS-domain transcription factor (Etv1) (O'Connor et al., 2009). In addition, the number of photostimulated neurons could be further restricted by expressing ChR2 only in a very small subset of neurons (Ako et al., 2011) or by using two-photon ChR2 stimulation methods (Rickgauer and Tank, 2009; Andrasfalvy et al., 2010; Papagiakoumou et al., 2010). Retrograde and anterograde trans-synaptic virus tracers may also be promising ways to yield more specific photostimulation (Miyamichi et al., 2010; Lo and Anderson, 2011). In the *in vivo* electrical recording, it was difficult to determine whether the recorded activity came from L5a or L5b neurons. Synaptic inputs to L5a and L5b neurons could be identified by combining whole-cell recording of target neurons in neocortical brain slices and photostimulation of ChR2-expressing axons from the other area (Petreanu et al., 2009; Hooks et al., 2013).

### Laminar connectivity patterns between the RFA and the CFA

We detected responses in the CFA when the neurons in L5b of the RFA were photostimulated in the ChR2 Tg mice. Although these results did not allow us to determine whether the upper layers of the RFA also projects to the CFA, our retrograde tracer experiments showed that axons originating from neurons in L5b of the RFA provided the dominant projections to the CFA.

By contrast, responses were absent in the RFA when the CFA was photostimulated in the ChR2 Tg mice, but were clearly present when the AAV-ChR2 mice were used. This difference demonstrated that while L5b of the CFA does not strongly project to the RFA, L2/3 and/or L5a of the CFA strongly does. These results are also consistent with our anatomical tracing experiments.

Synaptic projections that originate from L2/3 predominantly innervate the deeper parts of L5 and there is differentiation between corticostriatal and corticospinal outputs (Morishima and Kawaguchi, 2006; Brown and Hestrin, 2009; Anderson et al., 2010). Corticospinal neurons in L5b, which are the final outputs of motor cortical networks, receive excitatory inputs from corticostriatal neurons, but the corticostriatal neurons do not receive inputs from the corticospinal neurons (Kiritani et al., 2012). Therefore, information is unidirectionally transmitted from L2/3 neurons and L5a corticostriatal neurons to L5b corticospinal neurons within the RFA and the CFA. Our results indicate that L5b of the CFA does not strongly project to L5 of the RFA, but L5b of the RFA has strong projections to L5 of the CFA. Therefore, information that is processed in the upper layers of the CFA is transmitted to the RFA and the final outputs of L5b of the CFA are likely determined by integration of signals from the upper layers of the CFA and L5b of the RFA. Considering the asymmetric information flow between the RFA and the CFA (Figure 7), our results support the idea that the RFA is a higher motor area than the CFA (Neafsey et al., 1986; Rouiller et al., 1993; Smith et al., 2010; Tennant et al., 2011).

However, the laminar connection patterns among the supplementary motor cortex (SMA), the premotor cortex (PM), and the primary motor cortex (M1) in the primate are not hierarchical (Dum and Strick, 2005). The PM and M1 project to the spinal cord (Dum and Strick, 1991). Similarly, the corticospinal axons originate from both the RFA and CFA in the rat (Rouiller et al., 1993). Thus, it remains unclear whether the motor cortex network is comparable between rodents and primates. Nevertheless, the next step will be to find what signals are transmitted between the RFA and the CFA. Individual neurons that project to the other area can be retrogradely labeled (Sato and Svoboda, 2010). Furthermore, individual neurons that are activated in the remote area by ChR2 photostimulation could potentially be identified by expressing calcium-sensitive fluorescent molecules in these neurons. Therefore, two-photon calcium imaging of these neurons during a motor task like the lever-pull movement task (Hira et al., 2013) should reveal the characteristics of their activities during movement and allow us to understand how multiple motor output channels are organized by corticocortical interactions during elaborate movements.

### Conduction delay between the RFA and the CFA

We found that it took approximately 10 ms to transmit corticocortical information from the RFA to the CFA or from the CFA to the RFA. Given the dense anatomical projections between the RFA and the CFA, we were not surprised to observe direct synaptic connections between these cortical areas. However, the latency may be longer than expected for a direct monosynaptic connection. For example, neurons in the motor cortex respond to whisker stimuli approximately 8 ms later than neurons in the somatosensory barrel cortex in the mouse and this motor cortex response is thought to be mediated by direct synaptic projections from the somatosensory cortex (Ferezou et al., 2007). The distance between these areas is approximately 4 mm, which is longer than the approximately 2 mm between the RFA and the CFA. Therefore, it remains possible that indirect connections, such as cortico-thalamo-cortical connections, are responsible for signaling between the RFA and the CFA. It remains unknown whether the 10 ms delay that we observed between the RFA and the CFA is mediated by monosynaptic or polysynaptic connections.

Whatever its origins, this delay cannot be ignored because the temporal order of action potentials is important for information processing (Izhikevich, 2006) and spike timing-dependent plasticity (Froemke and Dan, 2002; Wolters et al., 2003). Thus, how the activity of the RFA and the CFA are temporally coordinated with these substantial conduction delays during movements could potentially be determined by simultaneous multi-electrode recording in both areas (Isomura et al., 2009).

## Summary

In this study, we combined *in vivo* ChR2 photostimulation mapping with *in vivo* electrical recording. This approach allowed us to demonstrate functional connections between the RFA and the CFA with conduction delay of approximately 10 ms in the mouse motor cortex. We found that the RFA receives strong functional projections from L2/3 and/or L5a, but not L5b, of the CFA, while the CFA receives strong functional projections from L5b of the RFA. Our results suggest that the neuronal activity that occurs in the RFA and the CFA during movement is generated through asymmetric reciprocal connections. The *in vivo* optogenetic mapping method will be useful to further clarify corticocortical functional connections.

### Conflict of interest statement

The authors declare that the research was conducted in the absence of any commercial or financial relationships that could be construed as a potential conflict of interest.
